# Design and Implementation of a Wireless Sensor and Actuator Network to Support the Intelligent Control of Efficient Energy Usage

**DOI:** 10.3390/s18061892

**Published:** 2018-06-09

**Authors:** Jesús Blanco, Andrés García, Javier de las Morenas

**Affiliations:** 1 AutoLog Group, Polytechnic School of Cuenca, University of Castilla-La Mancha, Cuenca 16071, Spain; 2 AutoLog Group, School of Industrial Engineering, University of Castilla-La Mancha, Ciudad Real 13071, Spain; Andres.Garcia@uclm.es; 3 AutoLog Group, School of Mining and Industrial Engineering, University of Castilla-La Mancha, Almadén 13400, Spain; Javier.delasMorenas@uclm.es

**Keywords:** wireless sensor and actuator networks, automated control, energy efficiency, energy management, energy saving

## Abstract

Energy saving has become a major concern for the developed society of our days. This paper presents a Wireless Sensor and Actuator Network (WSAN) designed to provide support to an automatic intelligent system, based on the Internet of Things (IoT), which enables a responsible consumption of energy. The proposed overall system performs an efficient energetic management of devices, machines and processes, optimizing their operation to achieve a reduction in their overall energy usage at any given time. For this purpose, relevant data is collected from intelligent sensors, which are in-stalled at the required locations, as well as from the energy market through the Internet. This information is analysed to provide knowledge about energy utilization, and to improve efficiency. The system takes autonomous decisions automatically, based on the available information and the specific requirements in each case. The proposed system has been implanted and tested in a food factory. Results show a great optimization of energy efficiency and a substantial improvement on energy and costs savings.

## 1. Introduction

The energy challenge is one of the most important issues for today’s society, and governments worldwide have to adopt strategic policies to face it. These actions involve important economic and structural consequences for daily life and need to be focused in solving problems with the available resources, to achieve a sustainable energetic future. The European Union (EU) is demanding strong actions from all member countries [1] on this respect. The “20-20-20” targets, within the 2020 climate and energy package (enforced by the Paris Agreement), envisage a significant reduction of greenhouse gas emissions, a substantial growth of renewable energy use, and major energy saving; all to be met by 2020. And for 2030, these actions will have to be improved further so as to reach an increase in energy efficiency of 27–30%. European governments have set up their own plans to meet these targets; for example, the Spanish Government has developed the “Energy Efficiency Action Plan 2017–2020”, to dictate ways to achieve these EU goals [2].

The EU focuses its actions on public transport and industry, due to their high potential for energy saving. However, all efforts in other sectors are also enforced as they can help achieving the specified targets. Some examples are energy labels for household appliances, or smart meters. Therefore, all economic sectors and activities must reduce energy consumption to perform tasks more efficiently, optimizing all available resources.

Renewable energies are also strongly supported in Europe; being wind the most significant of these sources. The WindEurope Association states that, in 2030, 31% of the generated energy will be produced by wind [3]. During 2016 wind was the principal source of renewable energy in Spain (47.3%), a country in which 40.8% of all electricity is produced by renewable sources [4]. Given their notable evolution, the peculiarities of these sources have to be taken into account, because they will be key for the future energetic framework. However, wind energy presents a great inconvenient as it depends on the intermittent nature of wind. This makes it prone to producing power peaks resulting in energy waste; a good management of available energy at each instant becomes especially relevant as a means to tackle this problem. Information and Communications Technologies (ICTs) are becoming crucial to improve the current situation by providing support to the efficient management of both energy demand and production [5].

The present work deals with the development of a Wireless Sensor and Actuator Network (WSAN) that provides the required support to an advanced management system for electric energy by supplying it with updated and reliable data as well as with a means to convey instructions. This is achieved through the use of a service network, that includes some features of the future unmanned intelligent plants proposed by [6]. The system contemplates different types of end users and provides an optimal management of their energetic necessities in a comprehensive, intelligent, and automatic way.

This paper is structured as follows: Section 2 presents the background in actions for solving the energetic problem, and the main technologies employed for it; Section 3 describes the different hardware and software parts of the proposed system, and their development; preliminary tests and applications are described in Section 4; and the results and analysis of the system performance are discussed in Section 5; finally, the last section presents the most relevant conclusions and future work.

## 2. Related Work

Industry is responsible for the use of a large quantity of the overall energy resources, and concerns on efficiency are driving actions towards optimizing energy consumption in industrial processes. To optimize this energy use, it is necessary to identify the specific actuation points in industrial plants: i.e., it is crucial to know the actual performance of the machinery, and all other energy consumers distributed within factories. WSANs present a great flexibility that makes them specially appropriate for applications requiring the acquisition and management of distributed data in almost any environment [7].

Along recent years, most of the developed work in this area has focused on Wireless Sensor Networks (WSNs) in the form of centralized networks for data collection. But the inclusion of actuation nodes is driving their evolution towards WSAN and opens the door to networks with intelligent decision-making capabilities that operate in a distributed way [8,9].

Some recent research has been carried out addressing all levels of WSAN technology: from the enhancement of the internal network organization, such as shown in [10] where different solutions for managing nodes and their interactions are analyzed and classified, or that in [11] where a system based on Deep Q-Network is proposed for mobile actor node control in WSANs; to the optimization of the nodes placement as proposed in [12], or the allocation of tasks to actuators found in [13]. All these ideas can be complemented with improvements in communications between nodes such as those presented in [14], where the use of a network over an IPv6 infrastructure is proposed. Also with the use of new algorithms to enhance the traffic forwarding capacity of nodes as described in [15], with proposals for reducing congestion and improving the network performance as in [16], a novel channel hopping methods for the exchange of data packages presented in [17], or an approach for enhancing the lossy link localization in [18]. But most of these works are based on simulations and few real results have been obtained from empirical environments, which may limit their direct applicability. Some additional work is being focused on reducing energy consumption of the WSANs; for example, in [19] PID control and fuzzy neural network algorithms are applied in nodes to control data transfer as well as node consumption. In [20], a thermal energy harvesting circuit for self-powered sensors is presented. In [21] several methods are proposed for choosing the actor node according to the task required to find the most efficient solution.

WSANs are suitable for applications in different scenarios, providing solutions to environmental challenges [22] such as energy saving for the reduction of greenhouse gas emissions. One of the most relevant applications in this context is the use of WSANs for smart buildings. For example, in [23] a WSAN is used for controlling the ventilation system in a building, and in [24] a self-powered WSAN enhanced the energy efficiency of the heat distribution system, through the use of energy harvesting techniques. As well as for new constructions, these systems can also improve the performance of old buildings, because they are easily adaptable and require little invasive installation. This is the case of [25], where a WSAN is employed to achieve savings in the energy consumption of the illumination system in a building, but the application of WSANs for energy savings does not only include big set-ups, as they can also be directly used to monitor and control the energy waste of electric appliances [26]. Or for providing support to more advanced control techniques such as in [27], where fuzzy control methods are applied over a WSAN in an industrial environment. Furthermore, a straightforward application is that it helps improve the control and management systems for air conditioning in buildings as shown in [28].

There are some ongoing global projects aiming at coordinating actions such as the development of specifications and standards for energy saving. A relevant example is the ZigBee^®^ Alliance [29], which produces innovative standards to enable manufacturers help customers with the deployment of the Internet of Things (IoT) through the establishment of Machine-to-Machine (M2M) wireless sensor and actuator networks. This allows the easy and cost-effective addition of intelligent new features that improve the efficiency, safety, security, reliability, and convenience of products and services. Some specific standards have been set for energy saving and energy efficiency: ZigBee Building Automation, ZigBee Smart Energy, ZigBee Home Automation or ZigBee Light Link.

Emerging examples that include some of the tools mentioned above are smart cities [30], which have a new energy management model, achieving a significant reduction of CO_2_ emissions. In this case, the use of energy storage systems with batteries is promoted, so that part of the available energy can be consumed afterwards for electric transportation, street lighting, or the air-conditioning of buildings. This kind of global actions may also comprehend complementary measures; e.g.,: the model used to enhance the demand response management of residential electricity consumers presented in [31], the use of models to improve the energy efficiency and usage in smart buildings, as proposed in [32,33], the application for reducing costs and enhancing energy savings with smart grids in railway lines presented in [34], or the appearance of platforms for the energy data analysis and management described in [35].

Energy saving is a topic in which all concerned parties must collaborate. However, efforts should not only be directed to the efficient generation of energy or to obtaining a larger amount of energy from renewable sources. The optimal solution would only be obtained by also looking into the transportation and consumption of energy [36]. Some efforts have been put on the distribution grids, such as that shown in [37], which presents a new methodology for the smart metering, monitoring and analysis of the grid, using Power Line Communications (PLC), low-voltage feeders and phase identification of smart electric meters. To face the increment of the share of renewables in the energy mix, novel techniques are proposed in [38] to avoid destabilizing the grid by providing a fast response to possible overloads. But, for an adequate control of the electricity supply grid in these cases it is also necessary to act at the demand-side [39,40,41] by complementary smart grid developments; e.g., solutions providing a fast response that matches consumption to supply peaks or differing energy usage to off-peak consumption periods.

Energy suppliers worldwide are beginning to implement new working policies and tools to contribute to energy saving through energy efficiency. Smart Meter Texas^TM^ is a State of Texas (USA) utility that allows users to view their daily electricity usage, and to employ data to understand their consumption patterns. It provides users with the capability to manage their energy usage, and the opportunity to reduce their costs [42]. In Spain, a new active billing system for small consumers is being implemented. It consists of a variation in energy costs, according to the time at which energy is consumed, thanks to the installation of smart meters. Red Eléctrica de España [4], the company responsible for the operation of the Spanish electric system, provides data about energy generation and consumption in real time. Consequently, this information allows the creation of new software tools that provide users with new ways of managing their energy consumption. All these new developments are bound to cause a change in society, which is key to achieving a sustainable energy future.

## 3. Proposal for Smart Energy Management

As has been established above, there is a great necessity for the optimization of energy consumption, especially for the case of electric energy, in all economic sectors. From the standpoint of the electricity source the aim is usually to enforce the use of renewable energy. While from an economic point of view, since energy costs represents a large percentage of the total costs of industrial processes, the aim is set at reducing the associated outlay.

This work presents a WSAN providing support to a new intelligent system for energy management and control that decides when devices should connect within a given range of time to optimize costs. Its operation is based on analysis and decision-making from real-time energy generation and consumption data, according to some conditions.

The overall system is exportable and adaptable to any industry or activity type and can be deployed for many specific applications, as described below.

### 3.1. System Overview

Many daily life activities at work, home or during leisure time could be carried out with considerable savings in energy consumption and, on most occasions, this can be made possible with only a very small effort. The proposed system manages, in an intelligent way, the energy requirements of end users to make them match the energy resources available at each instant. However, not all processes or tasks can undergo an energy restriction without further consequences, and it may not be always possible to disconnect elements at will. Therefore elements can be differentiated (in a factory, at home or at the working place) according to their energy requirements:

- Type 1: includes all machines or devices which need continuous and constant power; in general, machinery associated with continuous production processes.
- Type 2: devices with energetic inertia.
- Type 3: devices with some autonomy (e.g., those that can operate with batteries).
- Type 4: devices that can withstand a power outage at any time.

Given that not all jobs or activities use resources of the same type, the system will have to differentiate between processes. As, depending on the application and circumstances, some processes will allow more or less changes in their power supply. To begin with, it will be necessary to ascertain the energy requirements of the machines or processes and their required availability.

Furthermore, the system needs to obtain data from the electricity grid about energy generation, energy demand and costs in time. For this, real-time public data has to be collected over the Internet as provided by companies that operate the grid.

**Figure 1:**
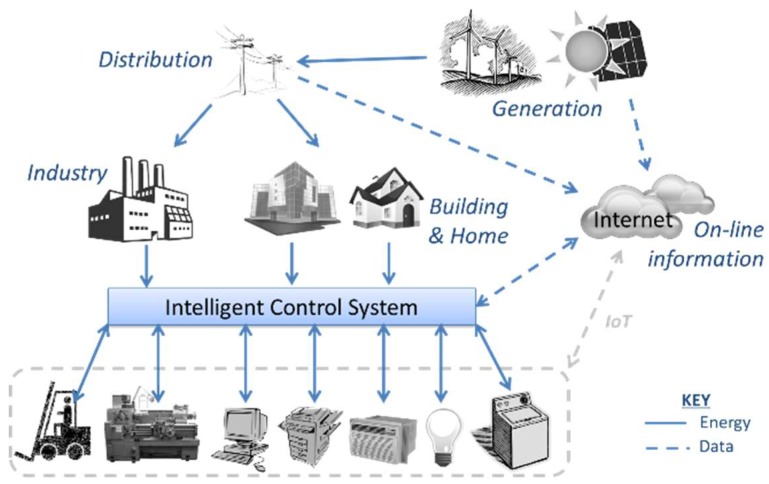
Structure and operation of the proposed system.

Through an adequate analysis of this information, the system becomes capable of automatically deciding when a process must be initiated, or a machine has to stop. For example, it can determine when it is possible to pre-warm an industrial oven with a minor energy cost so that it will be ready when needed; or it can decide when and how to charge some batteries, thus making the most of any possible surplus in generated energy. Furthermore, the system may benefit from the development of studies on ways to improve predictions on the most adequate time slots to consume energy based on cost and availability. This is why some learning capabilities have been incorporated on it from the outset. Figure 1 describes the framework in which the system operates, and the relationships between its different constituent parts.

The proposed system is divided into two parts: the hardware, which controls the power supply and captures information about real energy consumption and energy efficiency of the machinery; and the software, which captures information about the operation of electric networks, analyzes all collected data, and takes decisions that are executed by the hardware. Being the main subject of this paper, special emphasis will be put on the hardware development required for the WSAN being used.

### 3.2. Hardware Description

Since the system has been designed for a broad range of applications, the hardware part is a control and monitoring system, based on a WSAN, which can be used in any application. Furthermore, these networks allow the connection between machinery and the Internet, which integrates the system into the IoT framework.

The proposal represents the generic case in which the system is implemented independently of the pre-existing networks. In this case ZigBee^®^ technology was selected for the development of the required WSAN. Besides its outstanding evolution along the past few years, this selection is justified by the relevance of the following features for the system being proposed:

- The use of a frequency band that does not require an operating license: 2.4 GHz.
- Low complexity (low memory request).
- Low power consumption (devices can be powered by batteries).
- Mesh networks (this feature is not found in the majority of standards for wireless networks): self-creation, self-reorganization, and multi-hop routing protocol.

Initially, the system was designed with mains-powered nodes, but the deployment of additional battery powered nodes was found to be necessary to allow data collection in places without direct access to the general power grid. This is justified by the need for data collection on the environment, such as temperature and humidity conditions at specific points inside and outside refrigerated rooms, ovens or furnaces (measured with probes attached to nodes specifically located). It was also taken into account that the system normally operates indoors, although its performance would be better outdoors without walls and other obstacles producing signal attenuation.

**Figure 2:**
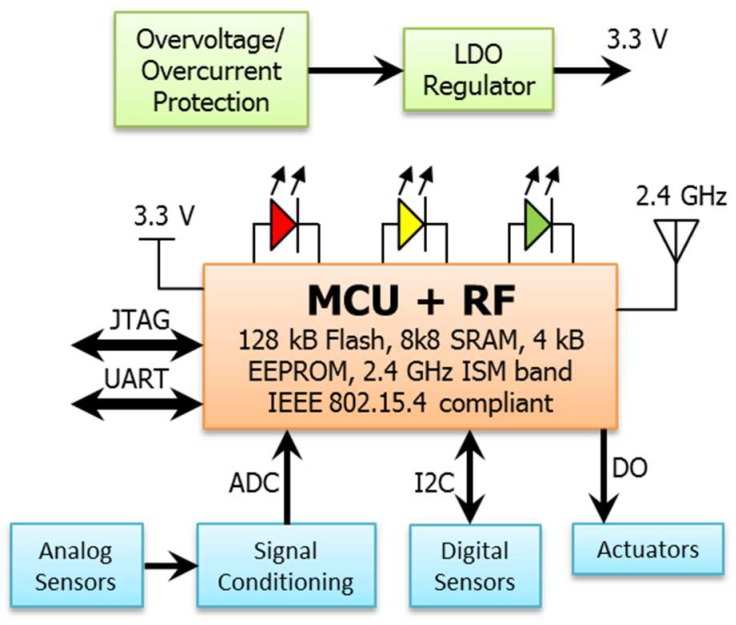
Block diagram of the coordinator/router nodes.

The wireless network consists of several nodes working as ZigBee routers. They have interconnection capabilities and are capable of managing network traffic, allowing the expansion of a previously established network, and/or adding redundancy. One of these nodes takes the role of network coordinator. Its main functions are: the network establishment and its interconnection with the user interface. Each node creates a coverage area for communications with other nodes. To decrease manufacturing costs, all nodes share a common hardware. Router nodes and coordinator node differ in the firmware and in the use they make of the Universal Asynchronous Receiver-Transmitter (UART) interface, apart from the number and type of sensors (Figure 2).

**Figure 3:**
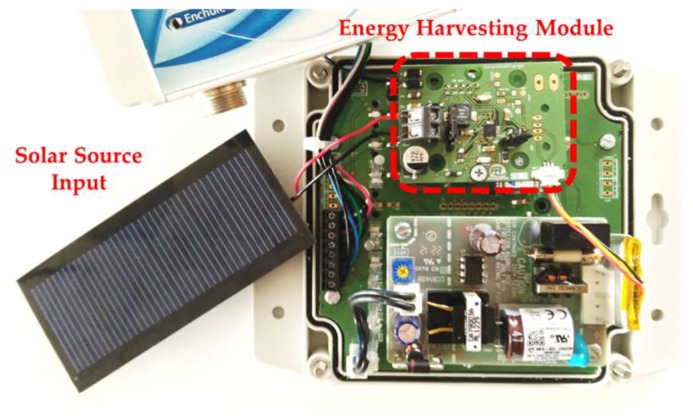
View of the PCB and components of a node with energy harvesting.

Nodes have been designed that can be supplied from several energy sources: directly from the grid (230 VAC), from a 5 VDC source or through a battery. An energy-harvesting module can also be included to charge the battery when collecting energy from sun or wind, as shown in Figure 3. The power supply stage provides the required 3.3 V at up to 500 mA. A linear Low Drop-Out (LDO) regulator was chosen, because it reduces the noise addition through the power signal. This is a very important consideration because the stability of the power supply is a key factor for the performance of the radio-frequency stage. The design of the power supply stage is completed with the inclusion of protection circuits, such as a voltage suppressor and current limiter.

In order to simplify the PCB design, a module [43] was chosen that integrates a microcontroller (MCU) with a Radio-Frequency (RF) transceiver, compliant with IEEE 802.15.4 at 2.4 GHz, a Low Noise Amplifier (LNA), and a Power Amplifier (PA) to provide a wide coverage range (over 1 km with direct line of sight outdoors). Its main features are: high reception sensitivity (−104 dBm); high power of signal throughout the link (up to 124 dB); up to 20 dBm of output power for transmission; reduced power consumption: (<6 μA in standby, 23 mA in receive mode and 50 mA in transmission mode); and a wide range of analog and digital interfaces.

With this set-up it is possible to incorporate a wide range of sensors to the nodes. To measure air temperature and humidity, a digital temperature and humidity sensor (−25 to 85 °C and %RH) was used that connects to the microcontroller through the I^2^C bus. An external analog temperature sensor (RTD) can be added to measure temperature inside the machinery (−50 to 150 °C). Its output has been DC-decoupled and amplified to accommodate it with the analog-to-digital converter of the microcontroller. To measure power consumption, the node includes a front-end to measure voltage, by means of which the line voltage is reduced, DC-filtered and DC-biased. It also includes a front-end to measure current (up to 200 A) that is also amplified, DC-decoupled and DC-biased. Both analog front-ends provide signals to the input of the analog-to-digital converter of the microcontroller.

**Figure 4:**
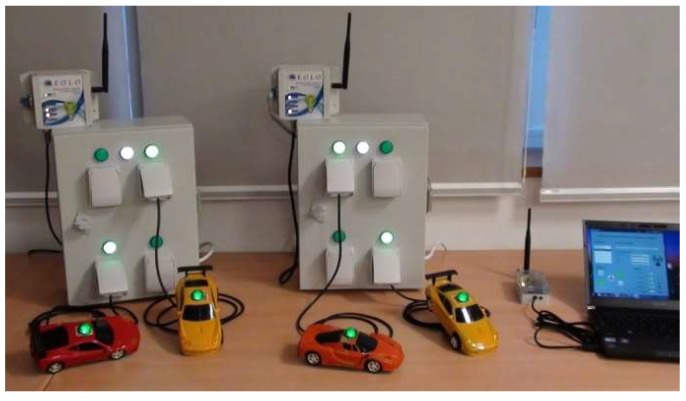
Laboratory tests of the final system before been released.

To work as actuators capable to switch on/off the power supply of the machinery, nodes are equipped with several solid-state relays that are coupled to digital outputs of the microcontroller. Figure 4 shows an operating example of the system.

#### Firmware

**Figure 5:**
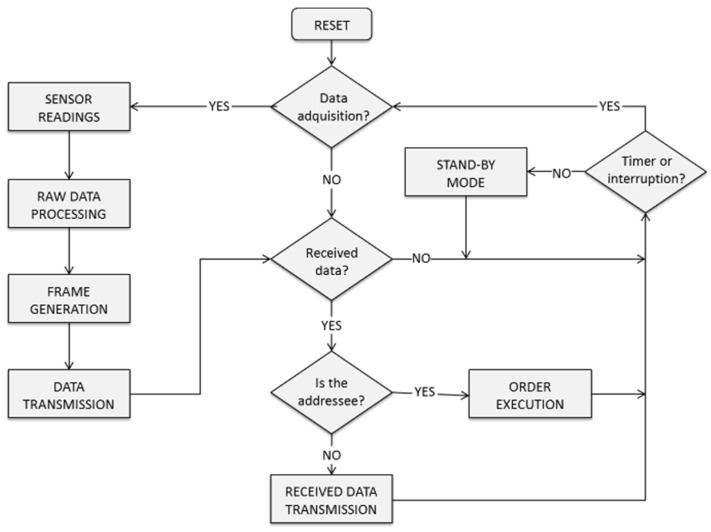
Firmware flowchart.

The WSAN performs the real-time energy consumption monitoring and control by using the required sensors and actuators at the nodes. Therefore, it acts as physical interface between the software part of the proposed system and the machinery whose performance is being optimized. Figure 5 shows a flowchart of the logic implemented at the WSAN nodes.

For energy efficiency monitoring, these devices take samples from their sensors periodically and these readings are duly processed before being transmitted. The required signal-conditioning is performed to optimize the measurement range, ensuring the coherence and reliability of the collected data. Furthermore, the hardware is always on the alert for specific requests, which may come from the software part of the system, for an appropriate control of the power supply to the attached machinery. When all of the above-mentioned tasks have been duly executed, these subsystems become idle and nodes enter in stand-by mode to reduce their energy consumption. This is particularly appropriate for the WSAN, where there may be nodes supported by batteries while performing energy harvesting.

**Figure 6:**
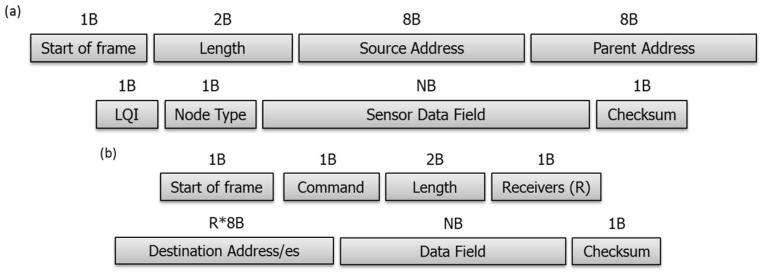
(**a**) WSAN sent frame format; (**b**) WSAN received frame format.

The WSAN has been implemented using a ZigBee PRO Stack, supplied by the manufacturer, which simplifies the firmware design and the interoperation with ZigBee devices of other manufacturers, if necessary. The information has been organized and packaged in data frames (Figure 6), which are the communication units used through the WSN. Nodes transmit and receive their corresponding data, but can also act as routers for frames related to other nodes.

### 3.3. Software Description

**Figure 7:**
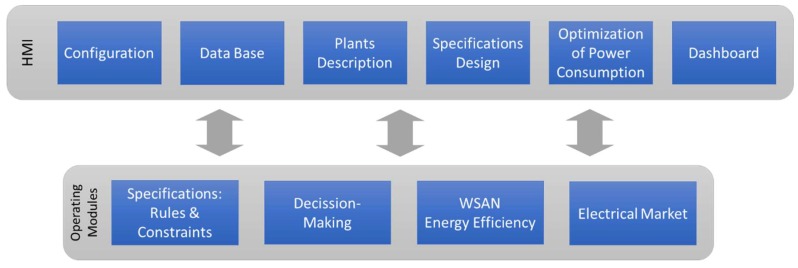
Software Architecture.

The proposed overall solution has a high-level logic platform, which is responsible for an adequate operation of the system as well as for providing decision taking capabilities. It resides in the Cloud, but it is possible to run it halfway between the Internet and local computers in partially isolated applications. Given its high complexity, this software platform is divided into several modules, which carry out a variety of related tasks. Their main functions are the energy efficiency analysis of the place where the system operates, the data collection of the electrical market, and the decision-making according to specific requirements of each application. This software also includes the user interface and data storage. The platform (Figure 7) has been developed using Java and a MySQL database.

#### 3.3.1. Energy Efficiency Analysis

One of the goals of the proposed system is to achieve optimal energy consumption. For this, it is necessary to know the actual consumption and efficiency of the installations and machines. So, the information provided by the hardware network (WSAN) is stored in a database. Afterwards, this information is analyzed, taking into account the specific features of the equipment and processes where the system will be installed. This makes possible the generation of a complete map of the energy efficiency of the place, through an energetic audit.

#### 3.3.2. Electrical Market Data Collection

The companies responsible for the operation of the electricity network provide information regarding the energy balance. They offer data about: the expected and actual energy demand; the real quantity of generated energy; and the energy generation structure, differentiating its origin. That is to say, it provides information about how much energy is generated by each source.

On the other hand, electric companies advertise the price of energy providing information that is available online. So, the system can access these web services for obtaining economic information, which is also used to operate. In most cases, information can be obtained in real time, allowing a quick and effective response of the system.

#### 3.3.3. Set-up of Specifications: Rules and Constraints

A set of rules and constraints must be stablished by end-users to summarize their requirements and specifications according to the desired use of energy in their facilities. Based on these guidelines, the system is capable of analysing the operating conditions to determine solutions that satisfy these requirements. This module is divided into two parts: the sub-modules for pre-sets and for operating energy rules.

The pre-sets submodule makes possible for users to introduce and configure the system operation according to their requirements: timetables and agendas, processes scheduling or special considerations, such as, the time in which a process needs to start, the temperature to pre-heat an industrial oven and when to do it, or the period in which a process can be switched off. On the other hand, the energy rules submodule is the tool that enables users to create and manage their energetic aims, that will be carried out when the specific conditions make it possible, e.g., that a process would only be carried out while renewable sources are producing a surplus of energy or that a process would only start when the price of energy goes under a certain threshold.

All these specifications are simple rules that, at this stage, do not require of a complex intelligent control as they can be easily evaluated. Furthermore, different rules can be grouped together and applied to the same process, thus making the setting of the specifications easier. The pre-set module also allows the user to establish levels of priority between rules to avoid conflicts during operation. Therefore, the control system can be kept simple as the strength of this solution lays in the accurate and timely data that is made available by the WSAN.

#### 3.3.4. Local Decision-Making

**Figure 8:**
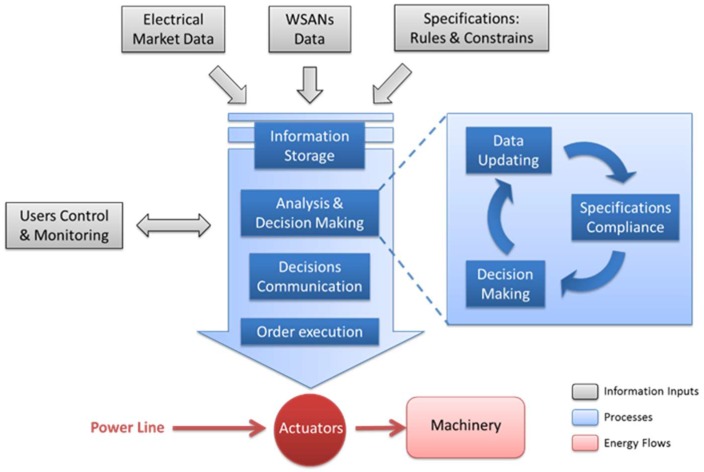
Logical architecture of the system.

This module is the main core of the software. It is based on a decision tree and supported by other parts of the system. Some of these parts provide it with the information inputs required for its operation: collected data from the electricity market; sensors data from the WSAN with information about energy consumption; and the specifications established by users through a group of constraints and rules to be met by the system. This module considers all collected data to make the best decision that ensures the compliance with the specifications set by users. As a result of the decision-making, the system manages the operation times of each device, and decides when and how the energy is consumed (Figure 8). The algorithm finds the best combination to carry out all the tasks, processing them with the lowest possible energy consumption. The system is continuously running and updating its input data so that any change at any instant can cause variations in the decisions.

The decision-making process starts with the analysis of the available data from the different sources (electrical market, WSAN and specifications set by users and machinery), at each moment. Based on this analysis, decisions are made to make the most of the available energetic resources at each moment. The algorithm is continuously doing these operations to evaluate whether it is possible to enhance the actual consumption scenario, while performing an on-line check of all conditions and applying the required conditions for them to be met.

For example, if six hours are needed to charge an electric forklift and there are twelve hours available to do it, the system will distribute the charging process throughout this time, to make the most efficient use of the available energetic resources and reduce its cost. Of course, being the main constraint, the system will make sure that the forklift gets charged by the specified time. This module also sends the required instructions to the control part (hardware) of the system, which executes them through the actuators at the nodes.

#### 3.3.5. Human Machine Interface (HMI)

**Figure 9:**
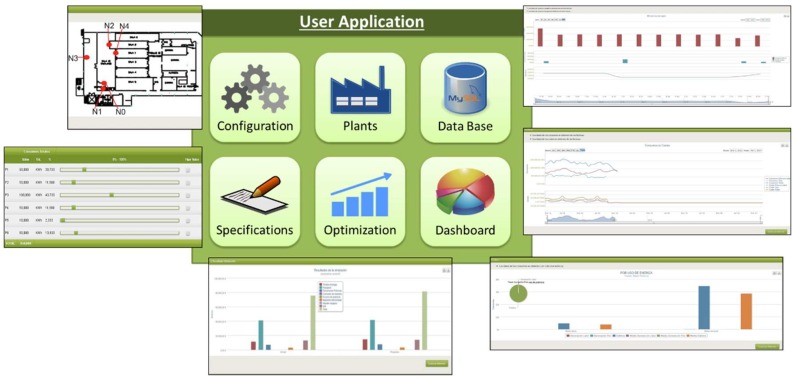
Graphical examples of the HMI.

In addition to collecting data or performing the decision-making, the interaction with users is a fundamental part of the system that makes possible the management of user roles, machines or energy providers. In this sense, an HMI has been developed that is supported by the above described modules. This high-level application is composed of different parts (Figure 9) that provide users with the required tools to manage and operate the system.

The *configuration part* is responsible for the general management of users, machinery and energy providers, as well as of the information related with energy contracts, consumption or other. Thanks to the *plants description part*, users can create tree diagrams of components including buildings, rooms, processes, machines, sensors and actuators, and can describe relations between them, such as assigning sensors and actuators to a machine. Later, these relations will be used in the energy design rules module. The *database part* supports the viewing and managing of all collected data from the WSAN and the Internet, as well as data generated by all software parts and modules. The *energy design rules part* makes possible for users the introduction all energetic preferences and specifications of machines and task scheduling. It is directly related with the specifications design module for system operation. The *optimization of the power consumption part* is supported by all software modules (with data being constantly updated by the WSN) to carry out simulations and show graphical representation of their results. Finally, the *dashboard part* shows numeric and graphical representations of actual consumption, costs and alarms, and enables users to monitor and check the proper operation of the plant.

## 4. Implementation and Applications

Once the corresponding preliminary tests had been successfully carried out to ascertain its correct operation, the system has been deployed in a scenario to evaluate its performance.

The performance of the system has been tested at a meat-processing factory, where there are several machines with high energy consumption. This factory covers 28,000 m^2^ and it has 300 employees. It processes 13 million kilos of meat per year, 30% of which are exports, with a billing that amounts to 55 million euros.

In this case, the WSAN was implemented with a coordinator and several router nodes, distributed according to coverage and functionality requirements. The nodes were attached to several slicing and packaging machines, cooling chambers, ovens, electric vehicles charging stations and air-conditioning machines. For the energy efficiency analysis of the plant, the system measured voltage and current at machinery, as well as environmental temperature and humidity in several workrooms and outdoors. For the power supply management of the machinery, the outputs of the nodes were connected to relays, which operated the power supply of specific equipment.

A set of tests was performed to the system once it had been deployed in the factory and before allowing it to start managing energy and controlling machinery. First, all sensors of each device were calibrated to provide reliable data from each sample. Later, the nodes were submitted to several coverage tests and it was ascertained that the indoors distance between nodes could be set at up to 230 m, as when nodes were further apart it was found that considerable amount of interferences distorted the signal. However, more than 600 m could be easily reached for outdoors scenarios. This big difference indoors-outdoors can be justified by the thick layers of isolation used for temperature controlled rooms. It was also concluded that no information packages were lost in the WSAN when nodes were located within the established distance from each other.

**Figure 10:**
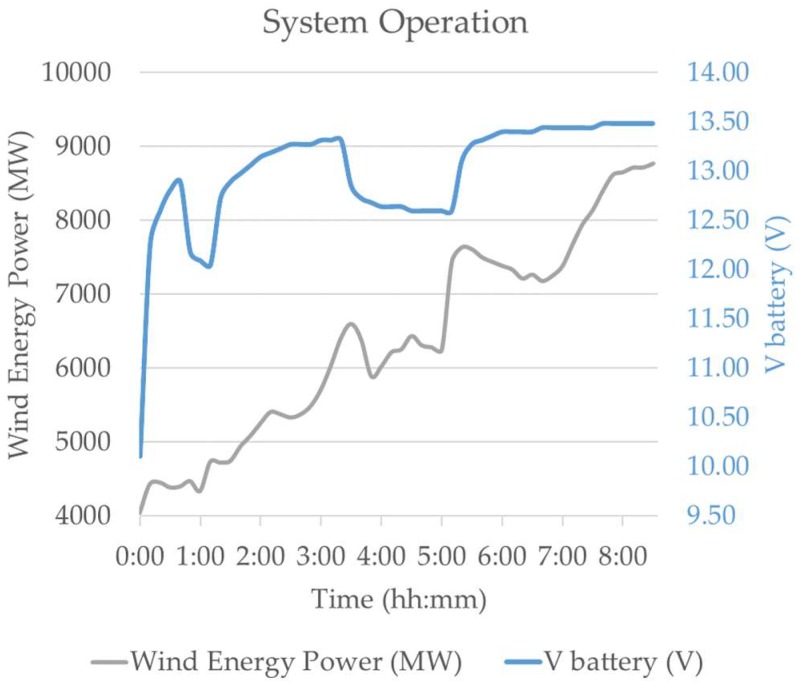
Charging process of a forklift and wind energy production as reported by the supplier.

An example of how the system works is shown in Figure 10, where a battery charging stage of an electric forklift was monitored and controlled. In this case, the charging process started when wind energy production was increasing and it was interrupted when the wind energy contribution decreased. The process started again each time that wind energy production increased and was interrupted when it decreased. But several minutes after five o’clock in the morning, the process was not interrupted when the wind energy contribution decreased because the time constraint was mandatory and it established that the battery had to be charged to a certain level (13.5 V) by 8:00 a.m. For this experiment, the battery voltage was monitored by one of the WSAN nodes and compared by the control system with the wind energy generation data as made available in real time by the energy supplier [44].

Apart from this type of industrial scenario the system, being low-cost and non-intrusive, can also be deployed in residences, where it can control the air-conditioning and the main electrical appliances, such as the oven, dishwasher, fridge and washing machine.

## 5. Results and Discussion

After several months of operation, it can be stated that the proposed system provides a new tool for the control of energy usage, capable to autonomously decide when each task can be performed under optimal conditions of efficiency.

The use of a WSAN makes the deployment of this system non-intrusive, as the data collection process does not require a direct connection to the elements being controlled. For the test case shown above, it was important for the management of the factory not to have any new system added that would tamper with the LAN and Wi-Fi being used in production. Furthermore, the data collection had also to be as non-intrusive as possible, for which the use of contactless probes for measuring values such as current, temperature and humidity was a key factor. While collecting other data such as voltage or battery charge is usually simple enough and often directly provided by existing equipment such as the battery charging stations (to which the nodes can be easily connected individually).

The factory where tests were performed is located at the east of Spain and by the sea. Therefore the environmental conditions of temperature and humidity are rather badly suited for meat processing. The results of the analysis provided by the proposed system helped improve the performance in some specific areas such as the processing rooms. However, the main overall result in this case was to ascertain that the high levels of energy consumption compared to other plants were mostly due to environmental conditions, which was duly justified to the investors.

The proposed system is also capable of providing control capabilities that act directly over the power supply of the different machinery without any modifications being required on them, only relays directly operating the sockets (Figure 4). Furthermore, this control is performed wirelessly and does not require any complex set-up operations.

The overall system provides the advantage of a fast adaption and use of peaks and excesses of energy that are produced regularly. This behavior is very important for electricity grids with a significant share of renewables, because of the intrinsic instability these sources produce. Therefore, the widespread deployment of the proposed system can easily contribute to make a greater share of renewable energy possible without destabilizing the energy grid.

On the other hand, from a business management point of view, the system helps taking full advantage of the collected information, making possible to choose the most appropriate policies or actions, and to decide new strategies to achieve a significant energy saving.

A further advantage of the system is that users can know in real time the energy state of their resources and processes. Also, thanks to the communication with electrical companies, it is possible to consume cheaper energy. Which will allow companies and users to reduce their energy consumption costs. The proposed system automates all actions and processes required for an efficient management of energy consumption.

Regarding the performance of the system in different scenarios, and taking into account the results obtained in the tests, it could be concluded that although its deployment can benefit a number of applications, its use can be specially justified for big consumers such as manufacturing plants.

## 6. Conclusions

The proposed system supposes an important improvement for the energetic sector and a strong impulse for Small and Medium Enterprises (SMEs). It provides a great progress in the pursuit of some priorities, defined by the EU, for future years; as are: the reduction of greenhouse gas emissions; the promotion of renewable energy use; and the use of new technologies and frameworks for energy sustainability. It may become a reference method for saving energy in daily activities at work or at home.

The proposal is a flexible and non-intrusive system that can be easily deployed and adapted to any application. It allows the optimization of available energy resources at each moment, managing the energy use of devices and machinery of the final users.

A future improvement of the system will be achieved through the addition of some new features, such as the incorporation of apps for mobile devices, which will provide easy management and monitoring of the systems anywhere. The system adapts seamlessly to new operational frameworks such as the Internet of Things (IoT), given that machinery and devices connect directly to web services to take distributed decisions (Figure 1).

It can be concluded that this work has presented the design and implementation of an intelligent automatic system to make the most of available energetic resources at each moment. Also that, by being built over a WSAN designed to capture data and provide control capabilities in a low-cost and non-intrusive way, the proposed overall system has gained a competitive advantage in being especially easy to deploy and use, even in complex environments such as running factories.
